# Factors associated with voice-related quality of life among patients with temporomandibular disorders^*^

**DOI:** 10.1590/1678-7757-2023-0296

**Published:** 2024-03-14

**Authors:** Rüdiger EMSHOFF, Magdalena ASTL, Aris Ioannis GIOTAKIS, Linus Christian HUPP, Andreas KOLK

**Affiliations:** 1 University Clinic of Oral and Maxillofacial Surgery Medical University of Innsbruck Innsbruck Austria University Clinic of Oral and Maxillofacial Surgery, Medical University of Innsbruck, Innsbruck, Austria.; 2 Bachelor Program Speech and Language Therapy University of Applied Sciences for Health Professions Upper Austria Linz Austria Bachelor Program Speech and Language Therapy, University of Applied Sciences for Health Professions Upper Austria, Linz, Austria.; 3 Department of Otorhinolaryngology, Head and Neck Surgery Medical University of Innsbruck Innsbruck Austria Department of Otorhinolaryngology, Head and Neck Surgery, Medical University of Innsbruck, Innsbruck, Austria.

**Keywords:** Orofacial pain, Temporomandibular disorders, Voice disorder, Anxiety disorder, Depressions

## Abstract

Research would be important for obtaining a better understanding of voice complaints among patients with temporomandibular disorders (TMD).

**Objective:**

The identification of predictors of voice disorders associated with TMD pain was made according to Diagnostic Criteria for TMD (DC/TMD) Axis I.

**Methodology:**

Functional limitations were measured using the Jaw Functional Limitation Scales for mastication (JFLS-M), jaw mobility (JFLS-JM), and verbal and emotional expression (JFLS-VEE). Patients also completed the Hospital Anxiety and Depression Scale (HADS). The primary outcome was social-emotional and physical functioning as indicated by scores on the Voice-Related Quality of Life (V-RQOL) questionnaire. Multiple linear regression was used to model the relationship between the domains on the V-RQOL questionnaire and scores on the HADS and JFLS after adjusting for age, gender, DC/TMD diagnosis, pain intensity, and time since pain onset.

**Results:**

The HADS-D (B=-1.15; 95% CI, -1.718 to -0.587; p<.001) and JFLS-VEE (B=-0.22; 95% CI, -0.40 to -0.06; p=.008) were significant predictors of scores on the V-RQOL questionnaire.

**Conclusion:**

Higher scores on depression measures and limitations in verbal and emotional expression could exacerbate voice problems among TMD pain patients. Future research should promote multidisciplinary treatments for TMD pain-related voice disorders.

## Introduction

Temporomandibular disorders (TMDs) include several clinical issues involving the temporomandibular joint (TMJ), muscles of mastication, and associated structures. These disorders are characterized by chronic pain and dysfunction.^1^TMDs have been estimated to have a prevalence of 3.7 to 12% in the general population. They are three to five times more common in women and are more frequently observed in middle-aged subjects.^2,3^ Additionally, an increased incidence rate of TMDs has been observed in women and young adults.^4^ Individuals affected by TMDs often have comorbidities such as headaches or neck pain, tinnitus, ear fullness and tension, dizziness, sensation of hearing loss, and speech and voice difficulties.^5-7^ Therefore, TMDs represent a common problem within the community.^8^ They are known to affect general health, psychological status, and social and economic well-being.^9,10^

Several studies have examined the onset of voice disorders in TMD patients,^6,7,11,12^ with the described prevalence ranging from 21% for loudness^11^ to 81% for voice handicap.^12^ For comparison, population-based prevalence rates of voice disorders were reported as 3–9%.^13^

It is difficult to diagnose voice disorders as a diagnosis requires a combination of thorough physical examination, a detailed history, a perceptual evaluation of the voice, videostroboscopy, and an analysis of aerodynamic and acoustic parameters.^14^ However, the impact of voice disorders on the social well-being, emotional state, physical health, and functional capabilities of patients can vary based on several individual factors. Hence, estimating the impact of a voice disorder on the overall quality of life of a particular patient is a crucial part of the clinical evaluation and may significantly affect the course of treatment.^15^

Some investigators have found significant negative correlations between TMD severity and vocal quality^11^ and voice-related quality of life (V-RQOL),^7^ and further a significant positive relationship between TMD severity and the total score of the voice handicap index (VHI).^12^ Meanwhile, others failed to show any correlation between TMD and vocal changes.^16^

Several authors have backed the idea that there is a connection between TMD and voice disorders, suggesting that certain TMD conditions could potentially lead to the gradual emergence of particular voice-related issues. These changes include decreased loudness,^11^ interference on vocal quality,^11^ alteration of voice resonance,^11^ reduced V-RQOL,^7^ greater vocal self-perception,^7^ and increased voice-related disability.^12^ However, the question of which TMD conditions make a significant biological contribution to the risk for voice disorders remains a point of controversy.^7,11^

While various potential factors related to TMD have been examined for their associations, as far as we are aware, there is a lack of available data for identifying predictor variables and developing diagnostic prediction models^17^ to estimate voice-related disorders in TMD patients. Conducting such research is vital for gaining a more comprehensive understanding of voice-related issues in individuals with TMD. Hence, the primary aim of this retrospective study was to utilize V-RQOL indicators to identify predictor variables for voice disorders in TMD patients experiencing pain.

## Methodology

### Study design, population, and inclusion and exclusion criteria

The study group was selected over a period of approximately one year and included 114 consecutive patients with unilateral or bilateral TMD pain due to arthralgia and/or myofascial pain. There were 103 females and 11 males, and the ages of the patients ranged from 18 to 71 years, with a mean age of 34.9 years. Patients with TMD pain who were referred for secondary care by their general dental or medical practitioner were eligible for this study. The subjects were informed about the study procedure, and written informed consent was received from them. This study was performed in accordance with the Declaration of Helsinki with respect to medical protocol and ethics and was approved by the local ethical committee (IMU IRB, Ref: 1205/2022).

Inclusion criteria were as follows: (1) the presence of a TMD diagnosis of uni- or bilateral arthralgia and/or myofascial pain assigned according to the DC/TMD;^18^ (2) an age from 18 to 75 years, (3) a pretreatment visual analog scale (VAS) pain intensity score > 10 mm, (4) pain lasting > 2 weeks and ≤ 5 years,^19^ (5) being ambulatory and able to be treated as an outpatient; and (6) being available for the study schedule. Exclusion criteria were as follows: (1) the presence of DC/TMD diagnosis of disc displacement without reduction and with limited opening (maximum assisted opening including vertical incisal overlap < 40 mm),^18^ (2) missing data for the relevant predictors, (3) an acute head or neck infection, (4) a diagnosis of collagen vascular disease, (5) a history of head or neck trauma, (6) a history of TMD treatment, and (7) a diagnosis of a debilitating mental or physical illness. Evaluation consisted of the collection of basic demographic information, self-report measures, and the patient’s medical history, followed by a physical examination. Clinical assessment was performed by a single well-trained clinician (RE) specialized in TMD and orofacial pain with more than 20 years of experience in this field. Measurements were made following a structured protocol.^20^

### Self-assessment instruments

Pain intensity was assessed using the VAS. Each subject rated their mean perceived severity of pain over the last month by using a 100-mm VAS ranging from 0 (no pain) to 100 (very severe pain). This scale has been used extensively in randomized trials and has shown good construct validity in comparison with other pain measures.^21^

Anxiety and depression were measured using the Hospital Anxiety and Depression Scale (HADS) to screen for clinically significant anxiety and depression in medical nonpsychiatric patients. HADS focuses on cognitive and emotional aspects of general anxiety and depression. It comprises seven questions to assess anxiety and seven questions to assess depression, and the responses for each item range from 0–3. Anxiety (HADS-A) and depression (HADS-D) are scored separately, and higher scores indicate a higher degree of distress. A score of >10 is considered a clinically significant disorder, whereas a score of >7 and ≤ 10 suggests a mild disorder.^22^

The Jaw Functional Limitation Scale-20 (JFLS-20) was applied to assess the functional status of the masticatory system. JFLS-20 is a 20-item organ-specific instrument covering three constructs: mastication (JFLS-M), vertical jaw mobility (JFLS-JM), and verbal and emotional expression (JFLS-VEE). The degree of limitation was evaluated on an 11-point numerical rating scale from (0) ‘no limitation’ to (10) ‘severe limitation.’ The scale can be used as an ordinal measure at a global level or for individual constructs. The total sum score for each construct in the JFLS-20 was calculated thus: mastication (0–60), vertical jaw mobility (0–40), and verbal and emotional expression (0–100).^23^ The JFLS has been validated in TMD patients with reliability coefficients of 0.82 for persons and 0.99 for items.^23^

The V-RQOL outcome measure was assessed via the German version^24^ of the V-RQOL.^25^ It is a valid 10-item questionnaire that measures the influence of voice disorders on quality of life. Each item is rated from 1 to 5 (from 1= “none, not a problem” to 5= “as bad as it can be”). The V-RQOL has a social-emotional domain and a physical functioning domain. Raw scores are summed to determine the total score (0–50), and an algorithm is used for summary scores, so that sum and domain scores range from 0 to 100, in which 0 indicates poor quality of life and 100 indicates optimal quality of life.^25^ The predictor (JFLS and HADS) and outcome variables (V-RQOL) were interpreted by the clinician (RE) and one investigator (MA) independently without knowledge of the results of the other assessments.

## Data analysis

Given that no preliminary data were available for effect size estimation, the number of subjects for this study was set on the basis of a medium effect size (Cohen’s f^2^=0.15), a significance level of 0.05, and a power of 0.80, and a total of nine variables were required for prediction modeling. As a result of the analysis using the G*power 3.1 software, the minimum number of samples was 114, indicating that this study satisfies the appropriate number of samples.

The independent sample t test and one-way analysis of variance (ANOVA) were used to compare the differences between subgroups. The bivariate Pearson correlation analysis for continuous variables was applied to determine the strength of association between V-RQOL questionnaire scores and those of the HADS and JFLS domains. Multiple linear regression was used to model the relationship between the outcome variable of V-RQOL questionnaire scores and those of HADS and JFLS after adjusting for age, gender, DC/TMD diagnosis, pain intensity, and time since pain onset. Each of the domains on the V-RQOL questionnaire was separately analyzed by stepwise linear regression to identify the specific JFLS and HADS items related to these subscales.

Cohen’s f^2^ was used as an appropriate measure for presenting the effect size within the multiple regression model. Cohen’s f^2^ was calculated using the following equation: $f^{2}=R^{2}/1-R^{2}$ , where R^2^ was the variance of the V-RQOL questionnaire scores explained according to the multivariable linear regression by HADS-D and JFLS-VEE together with the set of confounding factors. Cohen’s f^2^ index was applied to determine the magnitude of the effect size, in which f^2^≥0.02, f^2^≥0.15, and f^2^≥0.35 represent small, medium, and large effect sizes, respectively.^26^

Significance was indicated by P<.05. The PASW 28.0 (SPSS Statistics, IBM) package was used for statistical analyses.

## Results

**Figure 1:**
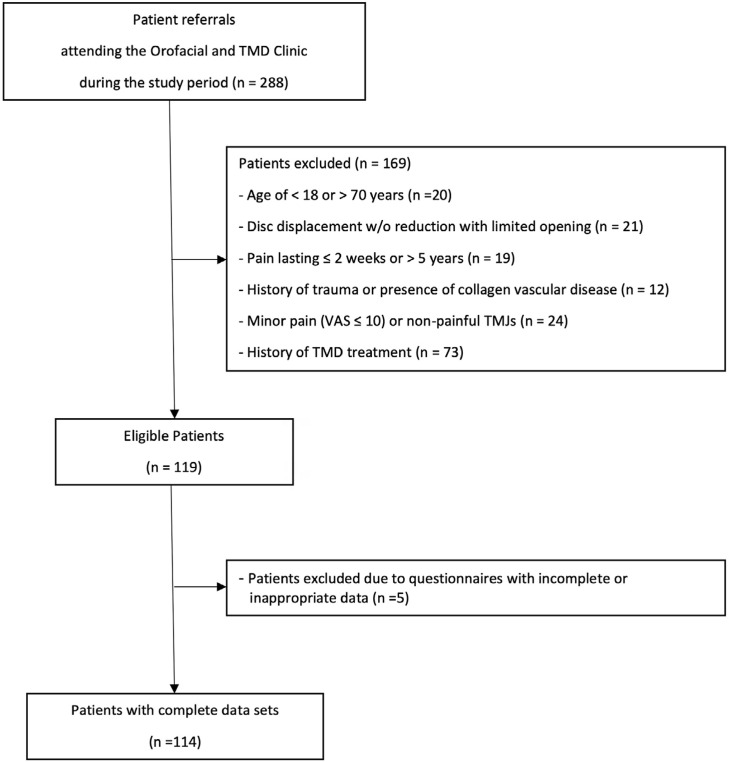
Flowchart demonstrating the selection process of the study population

Of the 182 consecutive referrals, 21 were excluded from this study due to the presence of a clinical diagnosis of disc displacement without reduction and with limited opening; 19, because of pain lasting ≤2 weeks or >5 years; and 12, due to a history of trauma or presence of collagen vascular disease. Moreover, 12 other patients were excluded as they either reported minor pain (measured as VAS≤10) or had non-painful TMJs with clinical findings of disc displacement with or without reduction. Out of the 119 patients that remained, we collected a total of 357 questionnaires. However, we had to exclude 11 questionnaires from five patients due to incomplete or inappropriate data. This left 114 TMD pain patients with 342 questionnaires (Figure 1).

Table 1 displays the characteristics of the eligible participants of this study. The mean age of the patients was 34.9± 3.5 years (range from 18 to 71 years), of whom 90% were female and 10% were male individuals.

**Table 1:** Patients’ characteristics (n=114)

Variables	Value
Age (years) (mean±SD)	34.9 (13.5)
Gender (n) (% female)	103 (90.4)
**DC/TMD diagnosis**	
Arthralgia (n) (%)	59 (51.8)
Myofacial pain (n) (%)	19 (16.7)
Arthralgia and myofascial pain (n) (%)	36 (31.6)
Pain intensity (mm) (mean ± SD)	45.1 (26.2)
Time since pain onset (weeks) (mean±SD)	78.2 (121.3)
V-RQOL (mean±SD)	88.0 (12.7)
Physical (mean±SD)	84.5 (14.6)
Social-emotional (mean±SD)	93.3 (13.9)
HADS score (mean±SD)	10.4 (7.4)
Anxiety (mean±SD)	6.5 (4.2)
Depression (mean±SD)	4.0 (3.9)
JFLS-20 score (mean±SD)	46 (14.2)
Mastication (mean±SD)	17.1 (10.2)
Mobility (mean±SD)	11.8 (6.9)
Verbal /emotion (mean±SD)	17.2 (15.8)

DC/TMD: Diagnostic Criteria for Temporomandibular Disorders.

V-RQOL: Voice-Related Quality of Life.

HADS: Hospital Anxiety and Depression Scale.

JFLS: Jaw Functional Limitation Scale.

Although no significant differences were found in mean physical functioning, social-emotional, and overall scores on the V-RQOL questionnaire between the pain intensity, time since pain onset, and DC/TMD groups (p>0.05), it was evident that subjects in the time since pain onset group III (pain duration, ≥ 2 years and ≤ 5 years) had markedly lower overall scores on the V-RQOL questionnaire than those in the time since pain onset in groups II and I (84.9 vs. 91.2 vs. 88.0, respectively) (Table 2-4 [Table t3] [Table t4]).

**Table 3:** V-RQOL Scores by time since pain onset (n=114)

	Time Since Pain Onset				
	TSO I	TSO II	TSO III		
	(≤6m)	(>6 m but <2 yrs)	(≥2 yrs but ≤5 yrs)	Total	
V-RQOL domains	(n=48)	(n=33)	(n=33)	(n=114)	p-value*
Social-emotional	94.3±11.2	95.4±11.5	90.0±18.8	93.3±13.9	0.242
Physical functioning	83.9±15.1	88.4±12.1	81.4±15.7	84.5±14.6	0.143
Total	88.0±12.2	91.2±9.4	84.9±15.6	88.0±12.7	0.129

V-RQOL: Voice-Related Quality of Life.

TSO : Time since pain onset.

*One-way analysis of variance. Adjustment for pairwise multiple comparisons was applied by the Bonferroni test.

**Table 2 t3:** V-RQOL Scores by VAS pain intensity (n=114)

	Pain Intensity#				
	I°	II°	III°	Total	
V-RQOL domains	(n=43)	(n=41)	(n=30)	(n=114)	p-value*
Social-emotional	92.9±15.0	94.2±11.6	92.8±15.6	93.3±13.9	0.885
Physical functioning	85.7±14.5	83.3±14.5	84.2±15.1	84.5±15.1	0.757
Total	88.6±13.6	87.7±11.2	87.7±13.7	88.0±12.7	0.93

V-RQOL = Voice-Related Quality of Life.

#Score levels on the 100 mm visual analog scale of pain intensity: I° (mild, ≤34), II° (moderate, >34 and <75), III° (severe, ≤ 75).

*Adjustment for pairwise multiple comparisons was applied by the Bonferroni test.

**Table 4 t4:** V-RQOL Scores by DC/TMD diagnoses (n=114)

	DC/TMD Diagnosis				
	Myofascial pain	Arthralgia	Myofasial pain & arthralgia	Total	
V-RQOL domains	(n=19)	(n=59)	(n=36)	(n=114)	p-value*
Social-emotional	93.1±16.5	94.8±10.6	91.2±17.1	93.3±13.9	0.474
Physical functioning	84.4±15.0	86.1±13.2	81.8±16.4	84.5±14.6	0.389
Total	87.9±15.0	89.6±11.0	85.6±13.9	88.0±12.7	0.332

V-RQOL: Voice-Related Quality of Life.

DC/TMD: Diagnostic Criteria for Temporomandibular Disorders. *One-way analysis of variance. Adjustment for pairwise multiple comparisons was applied by the Bonferroni test.

**Figure 2:**
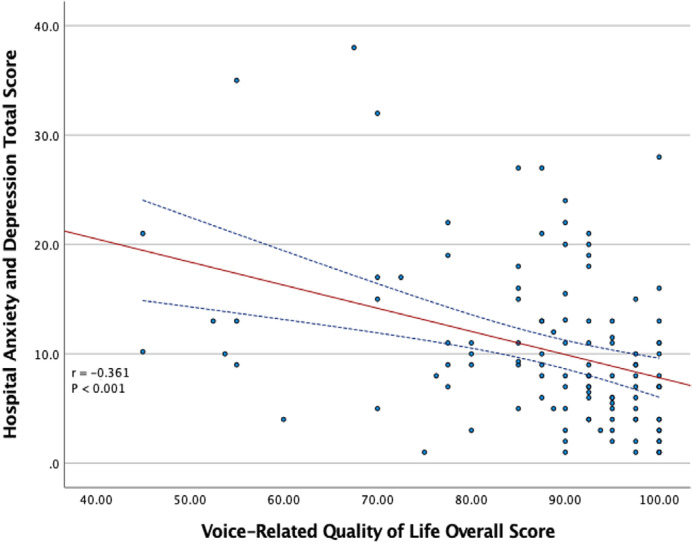
Scatter plot that represents the relation between the total score of the Hospital Anxiety and Depression Scale and the overall scores on the Voice-Related Quality of Life questionnaire. Pearson’s correlation, r= -0.361, p<.001

A significant difference was found in the overall scores on the V-RQOL questionnaire between patients with abnormal HADS-D and lower HADS-D scores (78.6±13.2 vs. 90.8±11.2, p=.004) (Table 5). Moreover, HADS total scores (r=−0.361, p<.001) were significantly and negatively correlated with the overall scores on the V-RQOL questionnaire (Figure 2).

**Table 5:** V-RQOL scores by HADS domains (n=114)

	HADS domains											
	Anxiety^**1**^ (n=114)				Depression^**1**^ (n=114)				Total^**1**^ (n=114)			
	I°	II°	III°		I°	II°	III°		I°	II°	III°	
V-RQOL domains	(n=80)	(n=15)	(n=19)	p-value*	(n=94)	(n=11)	(n=9)	p-value*	(n=47)	(n=21)	(n=46)	p-value*
Social-emotional	93.4±14.8	96.7±5.7	90.5±14.5	0.438	94.5±13.3	86.4±19.9	90.3±9.9	0.151	97.4±9.3^d^	91.1±17.0	90.2±15.5^d^	0.758
Physical functioning	86.0±14.1^a^	86.1±10.9	76.5±16.9^a^	0.033	86.7±12.8^b^	76.6±18.8	70.8±17.9^b^	0.001	90.0±10.4^e^	83.9±13.9	79.0±16.6^e^	0.014
Total	90.0±12.7	90.3±7.8	82.1±14.6	0.078	90.8±11.2c	80.5±18.4	78.6±13.2c	0.004	93.0±8.5f	86.8±13.0	88.0±12.7^f^	0.049

V-RQOL: Voice-Related Quality of Life.

HADS : Hospital Anxiety and Depression Scale.

*One-way analysis of variance. Adjustment for pairwise multiple comparisons was applied by the Bonferroni test.

^1^score levels of HADS domains: I° (normal, 1-7), II° (borderline abnormal, 8-10), III° (abnormal, > 10).

^a^p = 0.031. ^b^p = 0.004. ^c^p =0.029. ^d^p = 0.037. ^e^p = 0.001. ^f^p = 0.001.

**Figure 3:**
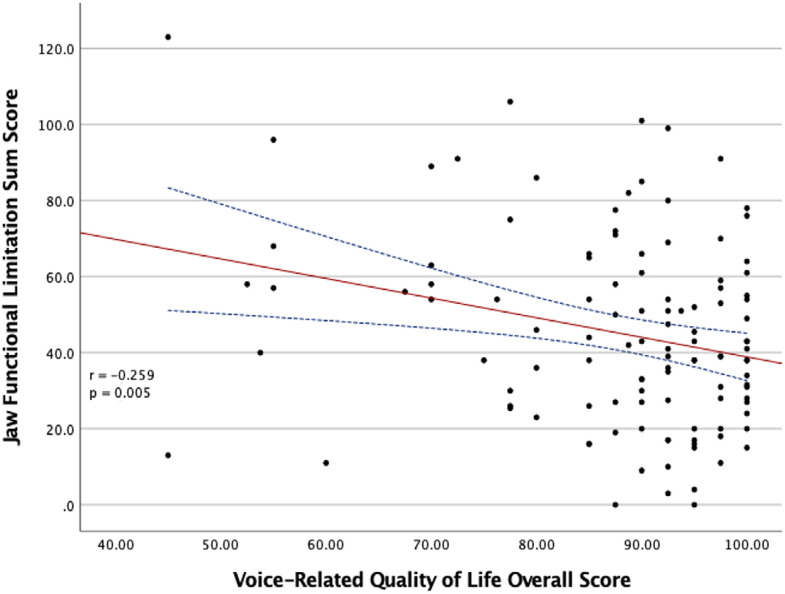
Scatter plot that represents the relation between the sum score of the Jaw Functional Limitation Scale and the overall scores on the Voice-Related Quality of Life questionnaire. Pearson’s correlation, r= -0.259, p=.005

Subjects with moderate JFLS-VEE scores had significantly lower scores on the physical functioning domain (74.7±17.1 vs. 86.1±13.6, p=.004) and the overall V-RQOL questionnaire (79.8±11.7 vs. 89.4±11.7, p=.005) than those with mild JFLS-VEE scores (Table 6). The total scores from the V-RQOL questionnaire displayed a negative correlation with JFLS sum scores (r=−0.259, p=.005), as illustrated in Figure 3.

**Table 6:** V-RQOL scores by JFLS domains (n=114)

	JFLS domains															
V-RQOL domains	Limitation in mastication^**1**^ (n=114)				Limitation in vertical jaw mobility^**2**^ (n=114)				Limitation in verbal and emotional expression^**3**^ (n=114)				Limitation in jaw function (total)^**4**^ (n=114)			
				p-value*				p-value*				p-value*				p-value*
	I°	II°	III°		I°	II°	III°		I°	II°	III°		I°	II°	III°	
	(n=73)	(n=41)	(n=0)		(n=62)	(n=52)	(n=0)		(n=98)	(n=16)	(n=0)		(n=93)	(n=21)	(n=0)	
Social- emotional	94.1± 13.0	91.9± 15.6	-	0.418	95.7± 10.3	90.5± 17.0	-	0.036	94.3± 12.5	87.3± 20.3	-	0.061	94.1± 12.6	89.9± 18.7	-	0.209
					93.1± 16.5				93.1± 16.5				93.1± 16.5			
Physical functioning	85.3± 16.5	83.0± 16.9	-	0.417	86.4± 12.7	82.2± 16.4	-	0.125	86.1± 13.6	74.7± 17.1	-	0.004	86.0± 13.7	77.9± 17.1	-	0.021
					93.1± 16.5	93.1± 16.5	93.1± 16.5		93.1± 16.5	93.1± 16.5	93.1± 16.5		93.1± 16.5	93.1± 16.5	93.1± 16.5	
Total	94.4± 13.0	93.9± 13.9	-	0.418	90.1± 16.5	85.5± 14.7	-	0.046	89.4± 11.7	79.8± 15.8	-	0.005	89.2± 11.7	82.7± 15.5	-	0.032

V-RQOL: Voice-Related Quality of Life. JFLS: Law Functional Limitation Scale. *One-way analysis of variance.

^1^Score levels of mastication: I° (0-20), II° (21-40), III° (41-60).

^2^Score levels of vertical jaw mobility: I° (mild, 0-13), II° (moderate, 14-27), III° (severe, 28-40).

^3^Score levels of verbal- and emotional expression: I° (mild, 0-33), II° (moderate, 34-66), III° (severe, 67-100).

^4^Score levels of Jaw Function (sum): I° (mild, 0-67), II° (moderate, 68-133), III° (severe, 134-200).

Linear regression analysis revealed that the independent predictors were HADS-D (B=−1.15; 95% CI, −1.718 to −0.587; p<.001) and JFLS-VEE scores (B=−0.22; 95% CI, −0.40 to −0.06; p=.008). Using the independent predictors obtained in the stepwise regression analysis, the following equation was obtained: V−RQOL=96.110−1.153× HADS-D −0.218×JFLS−VEE (R^2^=0.237, p<.001), in which the Cohens f^2^ was 0.31, representing a medium effect size (Table 7). Multicollinearity was tested using variance inflation factor with a value of 1.1123, indicating no multicollinearity.

**Table 7:** Multivariable model to predict V-RQOL scores among patients with a DC/TMD diagnosis of arthralgia and/or myofascial pain (n=114)

	B	S.E.	ß	t	P	95% CI
JDLS verbal/emotion	-0.218	0.080	-0.238	-2.715	0.008	-0.376 to -0.059
HADS depression	-1.153	0.285	-0.354	-4.039	<0.001	-1.718 to -0.587
Constant	96.110	1.761		60.706	<0.001	92.621 to 99.599
Cohen's f^2^ = 0.313						

V-RQOL: Voice-Related Quality of Life.

DC/TMD: Diagnostic Criteria for Temporomandibular Disorders.

JFLS: Jaw Functional Limitation Scale.

HADS: Hospital Anxiety and Depression Scale.

Cohen's f^2^: Effects sizes categorized as small (≥ 0.02), medium (≥ 0.15) or large (≥ 0.35).

For each of the JFLS-VEE items, separate regression analyses were conducted with the social-emotional and physical functioning domains of the V-RQOL questionnaire as the independent variables. The model was adjusted for factors including age, gender, time since pain onset, pain intensity, and DC/TMD diagnosis. The JFLS-VEE item “I can’t open my mouth wide enough to talk” was strongly and negatively associated with scores on both the social-emotional (p=.002) and physical functioning domains (p=.013), whereas the item “I can’t smile properly” was negatively associated with scores on the physical functioning domain (p = .025).

## Discussion

This study involving patients with a DC/TMD diagnosis of arthralgia and/or myofascial pain found no significant association between V-RQOL domains and the respective pain intensity and duration groups. However, it was observed that subjects with pain duration of ≥2 years and ≤5 years had markedly lower overall scores on the V-RQOL questionnaire than those in the other groups with shorter pain duration. While there are no available studies that specifically investigated the impact of orofacial pain intensity and duration on TMD patients’ V-RQOL domains, some studies have approached the relationship between musculoskeletal pain in the head, face, and neck regions and the respective V-RQOL domains in subjects with and without vocal complaints.^27,28^ According to the data obtained in the study by dos Santos, et al.^27^ (2019), a control group of non-voice-related professionals without vocal complaints presented significant negative correlations between (1) pain frequency in the “posterior neck,” “anterior neck,” and “masseter” areas and the scores on the V-RQOL questionnaire on the social-emotional domain and (2) pain intensity in the “submandibular,” “masseter,” and “temporal” areas and the scores on the V-RQOL questionnaire on the physical functioning domain. Furthermore, in another study involving a control group of subjects without vocal complaints, Ramos, et al.^28^ (2018) observed significant correlations between musculoskeletal pain and scores on the V-RQOL questionnaire, stating that the more frequent and intense the pain in the “anterior neck”, “submandibular”, and “temporal” region, the lower the V-RQOL on the physical functioning domain. The data from the aforementioned studies make it possible to conclude that vocal evaluations should include examinations of pain sensations in the submandibular and larynx regions (which are muscle regions near the vocal apparatus) as they may appear to be a symptom that results in functional dysphonia. This emphasizes the need for future studies to investigate the influence of specific pain conditions in the head, face, and neck regions on voice complaint characteristics and related consequences of voice complaints.

Our study results revealed that patients with abnormal HADS sum scores had significantly lower overall scores on the V-RQOL questionnaire than those with normal HADS total scores, whereas linear regression analysis adjusted for age, gender, time since pain onset, pain intensity, and DC/TMD diagnosis revealed that the HADS-D score was an independent predictor of scores on the V-RQOL questionnaire. Our study findings are in line with the results of previous investigations that demonstrate a positive relation between HADS sum and Voice Handicap Index scores in subjects with benign voice disorders.^29^ Nevertheless, there is no clarity about the particular function of psychogenic traits in various voice disorders or the association between the causal and predisposing factors in terms of developing voice disorders. These aspects have been considered in several studies showing that people with functional dysphonia (HADS-A, 61.5%; HADS-D, 35.4%),^29^ common voice disorders (HADS-A, 36.9%; HADS-D, 31.2%),^30^and benign voice disorders (HADS-A, 42.1%; HADS-D, 19.2%)^31^ suffer from increased levels of anxiety and depression. Currently, it is understood that voice and psychological disorders may coexist and that the direction of the association between voice disorder and psychological symptoms remains unclear.^29,32,33^ Longitudinal studies using validated and specific tools are required to clarify the temporal relationship between voice complaints and psychological factors.

The findings of this study, in which V-RQOL is considered as the dependent variable, represent the first evidence that individuals with TMD pain and depression are more likely to experience impairments in voice-related quality of life in comparison to those without depression. While there is no specific theory outlining the exact mechanism by which depression predicts voice-related issues, this study does confirm a significant association between psychological disorders and voice problems.^33^Nonetheless, the causality of the observed associations must be carefully interpreted because our retrospective study design does prohibit us to conclude whether depression and/or V-RQOL impairment developed before or after TMD onset. A window into this relation is the finding that a high percentage of our patients had concomitant chronic pain (57.9 %), anxiety (29.8 %), and/or depression (17.5%). This assertion is consistent with previous research that has documented clinically noteworthy rates of depression and anxiety among individuals with chronic TMD pain.^34^ On the other hand, voice function can also be recognized as a variable subject to alteration influenced by pain perception and functional challenges. This is due to the involvement of mandibular movements and structures that have a direct association with pain and functionality.^7,12^ For a more comprehensive examination of patients’ perspectives on the interplay of these factors, future studies should adopt a longitudinal design. These studies should encompass a larger sample size, including individuals with varying degrees of severity and duration of TMD-related pain conditions. Moreover, robust measures of psychological effects should be included to assess their influence on voice-related functions.

To the best of our knowledge, there is a lack of research examining TMD-related predictor variables of V-RQOL. However, such research would be important for obtaining a better understanding of voice complaints among TMD patients. This study used JFLS as this instrument has been validated for assessing the degree of limitation in three distinct constructs (mastication, mobility, and emotional and verbal expression)^23^ and has been applied in several studies for the assessment of global limitations associated with TMD.^35,36^ The data revealed that patients with moderate scores on limitations in vertical jaw mobility and verbal and emotional expression had significantly lower overall scores on the V-RQOL questionnaire than those with mild scores on limitations in vertical jaw mobility and verbal and emotional expression, whereas the overall scores on the V-RQOL questionnaire were negatively correlated with JFLS sum scores. Concerning the observed correlation between the severity of TMD pain-related limitations and the degree of quality of life impairment due to voice disorders, our findings may correspond to those of previous research reports describing TMD severity to be associated with the presence of changes in vocal quality,^11^ TMD severity and V-RQOL to be negatively correlated with one another,^7^ and TMD severity to show a significant positive relationship with the total score on the voice handicap index.^12^ However, results may prohibit direct comparisons as (1) these studies lack the use of evidence-based criteria for diagnosing TMD and assessing TMD severity and (2), in these studies, specific confounding variables were not considered in a multivariate design, i.e., studies failed to simultaneously consider the relevant demographic and clinical parameters. The fact that the numerous available dysphonia self-assessment questionnaires represent conceptual differences^37^ and that multiple-factor studies using valid instruments for assessing voice-related outcomes in TMD pain patients are unavailable indicates the necessity for further investigations to test the hypothesis that TMD pain patients with well-defined and clinically relevant characteristics do not differ from control subjects with regard to the presence of specific signs and symptoms of voice disorders.

The prediction model developed by multivariate regression analysis may be used as a risk prediction method for voice disorders and thereby provide valuable information for further clinical follow-up and treatment approaches. If patients are identified as being at a high risk by this prediction, clinicians may be able to control some important risk factors to reduce the risk of prolonged symptoms of voice problems. In practice, considering a hypothetical case involving a patient with TMJ arthralgia, in which HADS-D = 16 and JFLS-VEE = 90, the linear regression prediction equation is V−RQOL=96.110−1.153×16−0.218×90=58.042 . That is, the model predicts a V-RQOL score of 58, which denotes poor self-rated voice quality.^25^ Prognostic prediction models guide physicians throughout therapeutic management and have become a standard to aid clinical decision-making. Demographic, clinical, and imaging characteristics and specific test results are applied to derive these diagnostic models, thereby estimating the probability of having a particular outcome.^38^ With regard to the prognostic prediction model developed in this study, it is therefore advised that external validation is performed in ongoing studies before considering incorporating this model into clinical practice.^17,39,40^

This study provides a perspective on the contribution of single items in JFLS domains to the worsening of voice problems in TMD pain patients. While the single items in the mastication and vertical jaw mobility domains contributed no amounts to a change in voice-related score levels, a clear association of the emotional and verbal expression domain was evident for the “I can’t open my mouth wide enough to talk” items of the social-emotional (p = 0002) and the physical functioning V-RQOL items (p = 0013) and the “I can’t smile properly” item (p = 0025) in the physical functioning V-RQOL domain. Although there are some reports that approached the possible relations between TMD signs and symptoms and the occurrence of voice disorders,^7,11,12,40^ studies that addressed the role of clinical TMD-related single items in the definition of valid voice-related outcome parameters in a multivariate design are unavailable. In these studies, several factors have been mentioned to have the potential to influence the development of voice disorders, including muscular disequilibrium, pain in the chewing muscles, muscular hyperactivity, excessive tension in the cervical or orofacial region, and restricted mandibular range of motion. Future studies should clarify which general and biomechanical aspects may be associated with an elevated risk of developing voice disorders. Unlike a case-control study, only a well-controlled cohort study can establish how specific factors may contribute to these changes.

The methods of sample size estimation applied in this study may limit definitive conclusions. However, the use of Cohen’s f^2^ to estimate effect size might provide a standardized effect size estimate of the association between V-RQOL questionnaire scores and the variables of HADS-D and JFLS-VEE while considering confounders for V-RQOL questionnaire scores. Such standardized effect size estimate will enable researchers to compare findings across studies and benefit future researchers with sample size estimation.

### Study limitations

This research requires evaluation with certain limitations being considered. First, questionnaire items were assessed by just one observer, meaning that observer bias could have influenced data collection. Such possibilities could be mitigated by research that employed more than one observer and that used multiple centers for collecting data, comparing measurements, and undertaking statistical correlations. Second, the original self-report JFLS questionnaire was translated from English to German without describing the psychometric properties of a German-translated JFLS instrument for measuring global jaw functional limitation of the masticatory system. The original version translated to German gives no guarantee of similarities between measurement properties. Hence, further assessments and evaluations are needed to eliminate the negative effect of poor translation and insufficient cross-cultural validation, which may result in the inconsistency of data for measurement properties and consequently incorrect validity of the developed questionnaire.^41^ Third, the individual item scores from the HADS, JFLS, and V-RQOL questionnaires, which yield Likert-type ordinal rather than interval data, were treated as continuous variables. Although there is compelling evidence that parametric tests may be used with Likert-scale ordinal data,^42^ further studies should address the implications of using Likert-type data in multiple regression analysis, and the question whether it may result in substantial loss of information and biased regression coefficients. Fourth, the study cohort was relatively small, and equation accuracy generally varies with its application to additional samples. Thus, it may be necessary to create regression prediction equations that are specific to populations using more substantial cohorts that take into consideration ethnic-, gender-, and age-related elements that can contribute to variations in V-RQOL outcomes.

## Conclusion

Higher scores on depression measures and limitations in verbal and emotional expression could exacerbate voice problems among TMD pain patients. Future research should promote multidisciplinary treatments for TMD pain-related voice disorders.
